# Diverging pathomechanisms underlying collagen I-related and MBTPS2-related osteogenesis imperfecta: insights from patient-derived fibroblasts and iPSC-based modelling of bone

**DOI:** 10.3389/fendo.2026.1887145

**Published:** 2026-07-31

**Authors:** Pei Jin Lim, Giulio Marcionelli, Ceres Blättler, Silvan Gut, Marianne Rohrbach, Cecilia Giunta

**Affiliations:** 1 Connective Tissue Unit, Division of Metabolism and Children’s Research Centre, University Children’s Hospital Zurich and University of Zurich, Zurich, Switzerland; 2 Institute of Biomechanics, Department of Health Sciences and Technology, Zurich, Switzerland

**Keywords:** COL1-OI, collagen quality, induced pluripotent stem cell, MBTPS2-OI, osteogenesis imperfecta (OI)

## Abstract

Osteogenesis imperfecta (OI), characterised by low bone mass and bone fragility, is a heritable disorder with a heterogeneous genetic cause. Type I collagen is the most predominant type of collagen in the bone. Hence, the vast majority of patients with OI carry genetic variants in the genes that encode for type I collagen. However, a fraction of patients have defects in genes that either participate in collagen synthesis and maturation, in osteoblast maturation and functions including bone mineralisation, or with yet fully-understood mechanisms. An intriguing example is *MBTPS2*, a gene in which missense variants cause two non-overlapping clinical spectrums - either OI or a dermatological spectrum condition (IFAP/KFSD). Our work in the past decade aimed at molecular profiling of MBTPS2-OI using patient-derived fibroblasts. Here, we expand on this by generating induced pluripotent stem cells (iPSCs) from patient-derived fibroblasts and subsequently differentiated step-wise through the sclerotome and into osteoblasts. We also developed a Fiji-based image analysis pipeline to examine extracellular collagen misfolding and fibril organization in 2-dimensional (2D) fibroblast cultures *in vitro*, which will complement existing methods to qualitatively assess collagen. Together, qualitative assessment of type I collagen and iPSC-based *in vitro* bone modelling revealed differences in pathomechanisms underlying MBTPS2-OI and classical COL1-OI This showcases diverging pathologies underlying different genetic forms of OI and highlights the need for better molecular characterization of each genetic form to optimise approaches for patient management and treatment.

## Graphical Abstract

**Figure:**
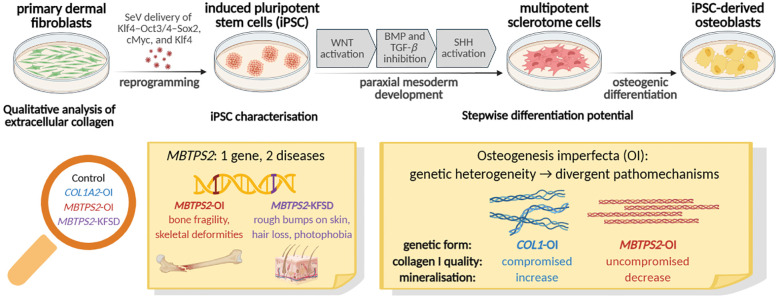
Summary of the experimental workflow and key findings. Primary fibroblasts were used for qualitative analysis of extracellular collagen and reprogrammed into iPSC to investigate osteogenic potential. The results collectively indicate that distinct pathogenic mechanisms underlie MBTPS2-OI and classical OI caused by glycine substitutions in type I collagen.

## 1 Introduction

Osteogenesis imperfecta (OI) is a rare inherited skeletal dysplasia characterised primarily by low bone mass, bone fragility and a lifelong predisposition to fractures, often after minimal or no trauma (1, 2). Its prevalence is commonly estimated at approximately one in 15,000–20,000 births (1, 3). Beyond recurrent fractures, OI is frequently associated with short stature, long-bone bowing, scoliosis, and a constellation of extra-skeletal manifestations including blue or grey sclerae, dentinogenesis imperfecta, hearing loss, muscle weakness, cardiovascular complications and respiratory insufficiency (1). In severe forms, progressive skeletal deformity and a compromised respiratory system may substantially contribute to morbidity and mortality. This systemic manifestation in OI reflects the importance of connective tissue integrity in health. In the classical Sillence framework, OI clinical severity spans a wide spectrum, ranging from mild forms with relatively preserved growth and mobility to progressively deforming disease and perinatal lethal presentations. Current practice increasingly combines phenotypic description according to Sillence nosology with the underlying causal gene (4). Importantly, the severity of OI varies widely not only between different genetic forms but also among individuals carrying similar variants (5–7). This variability suggests that disease manifestations result from a combination of the primary molecular defect and secondary alterations in bone development, extracellular matrix (ECM) organization, and cellular stress responses.

OI was historically regarded as a disorder of type I collagen, the principal organic component of bone matrix (8). 80-90% of patients with OI harbour pathogenic variants in *COL1A1* or *COL1A2*, which encode the α1(I) and α2(I) chains of type I collagen, respectively (1, 6). Variants in these genes may cause either quantitative or qualitative defects. Haploinsufficiency mutations lead to nonsense-mediated decay of the mRNA causing quantitative defects of structurally normal collagen and is often associated with mild disease presentation. Qualitative defects involve structural abnormalities associated with substitution of glycine or other highly conserved amino acid residues within the collagen triple helix region (6). The genetic landscape of OI has broadened substantially over the past two decades. In addition to *COL1A1* and *COL1A2*, numerous genes have now been implicated in recessive, dominant, and X-linked forms of OI, including genes involved in collagen post-translational modification, folding, intracellular trafficking, secretion, ECM assembly, mineralisation, and osteoblast differentiation; the heterogeneous genetic causes and pathology underlying OI are summarised in several recent reviews (2, 4, 9). This expanded view has shifted the concept of OI from a purely structural collagenopathy to a group of disorders affecting several interconnected pathways required for normal bone formation and homeostasis.

A particularly unexpected addition to the OI disease spectrum was *MBTPS2* (10), a gene already well established as the cause of several X-linked genodermatoses, as summarised in a recent review (11). *MBTPS2* encodes an essential membrane-bound transcription factor peptidase, site-2 protease (S2P), that mediates regulated intramembrane proteolysis to release the active cytosolic domains of several membrane-tethered transcription factors (10–12). Among its characterised substrates are the sterol regulatory element-binding proteins (SREBPs) which regulate cholesterol and fatty-acid biosynthesis, and activating transcription factor 6 (ATF6) which is a major mediator of the unfolded protein response and endoplasmic reticulum (ER) stress adaptation. S2P also activates members of the cAMP-responsive element-binding protein (CREB3) family, including CREB3L2/BBF2H7 (Box B-binding factor 2 Human homolog on chromosome **7**) and CREB3L1/OASIS (Old Astrocyte Specifically Induced Substance), which are recognised for their importance in chondrogenesis and osteogenesis, respectively (13–16). Through these substrates, MBTPS2 sits at the intersection of lipid homeostasis, ER proteostasis and cellular stress responses, and tissue-specific differentiation programs. These functions are highly relevant to skeletal biology because bone-forming cells must sustain intense secretory activity to produce ECM proteins including collagens. Impaired MBTPS2 function is therefore predicted to affect not only generalised cellular stress responses but also the production of a healthy functional bone matrix.

Missense variants in *MBTPS2* causing a loss-of-function of S2P were first identified in ichthyosis follicularis, atrichia and photophobia (IFAP, OMIM 308205, characterised by non-inflammatory thorn-like follicular keratosis, absence of hair and abnormal sensitivity to light) syndrome, with or without additional symptoms of BRESHECK (Brain anomalies, Retardation, Ectodermal dysplasia, Skeletal anomalies, Hirschsprung disease, Ear/eye abnormalities, Cleft palate, and Kidney dysplasia) syndrome, in keratosis follicularis spinulosa decalvans (KFSD, OMIM 308800 characterised by widespread hyperkeratotic follicular papules), and in Olmsted syndrome (OMIM 300918, characterised by thick, hardened skin on the palms and/or soles and painful keratotic plaques around the mouth, nose and eyes) (17–19). The later discovery that missense loss-of-function variants in *MBTPS2* can also cause an X-linked recessive form of OI affecting males (OMIM 301014) was therefore particularly striking, as it unexpectedly connected a gene previously associated with ectodermal pathology to bone fragility and further expanded OI beyond the traditional autosomal dominant and recessive inheritance patterns (10, 20). In affected individuals, mutant S2P is expressed but exhibits impaired regulated intramembrane proteolysis of substrates including OASIS/CREB3L1, ATF6, and SREBP, with downstream evidence for reduced type I collagen secretion, abnormal collagen cross-link (10) and perturbed fatty acid metabolism in fibroblasts *in vitro* (21). These rare conditions collectively emphasise the broad requirement for *MBTPS2* in developmental homeostasis. To date, five unique missense MBTPS2 variants have been described in patients with OI, none of whom present with symptoms associated with IFAP/KFSD/Olmsted syndrome: p.Glu172Asp (20), p.Leu455Gln [unpublished communication], p.Asn459Ser [(10, 22), unpublished communications], p.Leu505Phe (10) and p.Gly512Arg (23). p.Glu172Asp was reportedly observed in a case of IFAP syndrome (24) but unpublished personal communications with the variant depositors revealed skeletal features in the proband which is more reminiscent of OI.

Loss-of-function variants in *Mbtps2* lead to embryonic lethality in hemizygous male mice, while heterozygous female mice fail to reproduce the hallmark skeletal manifestations of MBTPS2-OI, including alterations in the mass, morphology or structural properties of the bone (25) but instead displayed osteochondral abnormalities resembling an early osteoarthritis (OA) phenotype not seen in MBTPS2-OI patients. *mbtps2* crispant zebrafish display severe craniofacial abnormalities and a survival rate of less than 10% at 30 days post fertilization (26). This phenotype contrasts with the clinical presentation of MBTPS2-OI patients, who predominantly display deformities in the sternum and long bones (10) rather than profound craniofacial defects. The high mortality observed in both mouse and zebrafish *Mbtps2* loss-of-function models, together with their phenotypic divergence from patients with MBTPS2-OI, limits their utility for investigating post-embryonic skeletal development, bone remodelling, and long-term disease progression.

Given the abundance and importance of type I collagen in maintaining the structural integrity and biomechanical properties of the bone, and its involvement in the pathology of OI, the assessment of collagen I quality is crucial for investigating OI. This can be achieved by several methods, each with their strengths and weaknesses. Steady state analysis is a well-established method for analysing *in vitro* collagen production. By labelling of the newly synthesised collagen proteins followed by sodium dodecyl sulphate polyacrylamide gel electrophoresis (SDS-PAGE), the method allows for semi-quantitative as well as qualitative assessment of the collagen I molecules based on their molecular weight which informs about processing of procollagen to mature collagen, and their migration pattern on the gel which gives insights into their over- or under-modification (27). However, since the analysis requires the proteins to be extracted from the cells and/or media, steady state analysis does not evaluate the spatial organization of the deposited extracellular network (28). Circular dichroism (CD) spectroscopy represents another technique for assessing collagen structure. Healthy collagen I possesses a characteristic spectral signature and deviations from this signature indicate alterations in its secondary structure and reflect structural defects (29). When combined with rheological methods, CD spectroscopy further allows assessment of collagen fibrillogenesis and denaturation (30). Similar to steady state analysis, CD spectroscopy does not provide information about the spatial organization of the collagen fibrils. In contrast, second harmonic generation (SHG) microscopy offers insight into collagen fibril organization by allowing detailed imaging at submicron resolution of biological tissue. This enables the investigation of pathological effects of diseases on the ECM. However, SHG is a costly setup and delivers only structural images that still requires subsequent quantitative image analysis.

Molecular characterisation and profiling of OI by application of a variety of methods such as collagen biochemical analyses, micro-computed tomography and biomechanical tests, and transcriptomics, proteomics and metabolomics analyses have been performed on primary dermal fibroblasts (10, 21, 31–33), osteoblasts (10, 34, 35) and bone explants (36, 37). The invasive nature of bone sampling makes the availability of bone explants and osteoblasts extremely limited for examining OI pathology, and even more so in the rarer genetic OI forms. Fibroblasts, while being a great cell model due to the relative ease of taking biopsies and their ability to produce ECM proteins including type 1 collagen, cannot fully recapitulate bone physiology. To overcome this limitation, reprogramming of primary fibroblasts or peripheral blood mononuclear cells into induced pluripotent stem cells (iPSCs) and subsequent differentiation into cells of the skeletal system have been performed to allow better investigations of OI pathology (38–42). iPSCs offer a versatile platform for modelling skeletal development and disease, as they can be differentiated into osteoblasts capable of producing and mineralising bone-specific ECM. Different protocols aim to recapitulate embryonic skeletal development, particularly axial bone formation from sclerotome derived mesenchyme through stepwise modulation of key signalling pathways. iPSCs can be directed to differentiate into chondrocytes that undergo hypertrophy and transition into osteoblasts and osteocytes under defined culture conditions, enabling faithful modelling of bone development and skeletal dysplasia (38, 43). Alternative methods, including embryoid body-based differentiation (44), retinoic acid-enhanced osteogenesis (45) and co-culture of osteoblasts and osteoclasts involved in bone remodelling (44) provide alternative *in vitro* approaches for modelling skeletal development with varying degrees of developmental fidelity.

We previously found by untargeted transcriptomics and targeted metabolome analyses that the expression of genes involved in lipid homeostasis and the relative abundance of fatty acids were perturbed in MBTPS2-OI patient-derived fibroblasts (21). Here, we expand our characterization of MBTPS2-OI by investigating the quality of extracellular type 1 collagens produced by primary fibroblasts using an image analysis pipeline and by modelling embryonic skeletal development using fibroblast-derived iPSCs to further investigate the pathomechanisms underlying *MBTPS2*-OI.

## 2 Materials and methods

### 2.1 Primary dermal fibroblasts

Healthy fibroblasts were obtained commercially (Gibco, C-004-5C, lot 2181181) or from foreskin fibroblasts of anonymised donors. Skin punch biopsies were obtained from patients with COL1-OI, MBTPS2-OI or MBTPS2-KFSD to establish primary skin fibroblasts cultures. The donor characteristics are summarised in **Table 1**. Cells were cultured at 37 °C and 5% CO_2_ in Dulbecco’s Modified Eagle’s Medium (DMEM, Gibco, 31966-021) supplemented with 10% foetal bovine serum (Gibco, 10270-106), and antibiotics (Gibco, 15240-062) compromising of 100 U/ml penicillin and 100 µg/ml streptomycin. This study was conducted according to the Declaration of Helsinki and approved by Swiss Ethics Committee (KEK-ZH-Nr. 2019-00811).

**Table 1:** Characteristics of fibroblast donors.

Fibroblast ID	Group	iPSC generated	Source/genetic variant
C1	Healthy control	✓	Gibco, C-004-5C, lot 2181181
C2	Healthy control		–
C3	Healthy control		–
C4	Healthy control		–
C5	Healthy control		–
GLY1	COL1-OI, glycine substitution		*COL1A1* c.976G>A, p.Gly326Arg
GLY2	COL1-OI, glycine substitution		*COL1A1* c.1648G>A, p.Gly560Ser
GLY3	COL1-OI, glycine substitution		*COL1A1* c.1648G>A, p.Gly560Ser
GLY4	COL1-OI, glycine substitution		*COL1A1* c.3496G>T, p.Gly1166Cys
GLY5	COL1-OI, glycine substitution		*COL1A2* c.568G>A, p.Gly190Arg
GLY6	COL1-OI, glycine substitution		*COL1A2* c.1378G>A, p.Gly460Ser
GLY7	COL1-OI, glycine substitution	✓	*COL1A2* c.2314G>C, p.Gly772Arg
GLY8	COL1-OI, glycine substitution		*COL1A2* c.2314G>C, p.Gly772Arg
HAP1	COL1-OI, predicted haploinsufficiency		*COL1A1* c.2144delC, p.Pro715Leufs*51
HAP2	COL1-OI, predicted haploinsufficiency		*COL1A1* c.3076C>T, p.Arg1026*
HAP3	COL1-OI, predicted haploinsufficiency		*COL1A1* c.3882_3883delGA, p.Glu1294Aspfs*3
MBTPS2-OI1	MBTPS2-OI	✓	*MBTPS2* c.1376A>G, p.Asn459Ser
MBTPS2-OI2	MBTPS2-OI	✓	*MBTPS2* c.1515G>C, p.Leu505Phe
MBTPS2-KFSD	MBTPS2-KFSD	✓	*MBTPS2* c.1523A>G, p.Asn508Ser

### 2.2 Qualitative assessment of collagen type I

Fibroblasts were plated on 6-well plates containing three glass coverslips per well at 400’000 cells per well. After four hours, the medium was replaced with 2 ml filter-sterilised macromolecular crowding (MMC) medium defined as DMEM (Gibco, 31966-021) supplemented with 37.5 mg/ml Ficoll PM70 (Sigma, F2878), 25 mg/ml Ficoll PM400 (Sigma, F4375), 0.5% FBS (Gibco, A5256701), 50 μg/ml Vitamin C (L-ascorbic acid, Sigma, A4544) and 1× antibiotics comprising 100 U/ml penicillin and 100 μg/ml streptomycin (Gibco, 15140-122). In the subsequent three days, a stock of 5 mg/ml Vitamin C was prepared fresh daily and 20 μl of the stock solution was added per well without replacing the MMC medium to achieve a final concentration of 50 μg/ml fresh Vitamin C daily to promote ECM protein synthesis and deposition. The next day, cells were washed three times with Dulbecco’s Phosphate Buffered Saline (DPBS, Gibco, 14190144), fixed with 4% paraformaldehyde for 15 minutes at room temperature and washed three times with DPBS. The fixed samples were stored in the dark at 4 °C until they were used for staining.

The coverslips were incubated with blocking buffer (1% bovine serum albumin (BSA, Sigma, A3912), in DPBS) for 60 minutes. To probe for collagens, co-staining of type I collagen and misfolded or unfolded collagens was performed as follows: 20 μM collagen hybridizing peptide conjugated to Cyanine3 (R-CHP, 3-Helix) solution was mixed with DPBS, heated at 80 °C for 5 minutes and cooled for 1 minute on ice. A primary antibody specific for type I collagen (**Table 2**) was added to the cooled R-CHP solution at a dilution of 1:500. The resulting solution was applied to the coverslips and incubated at 4 °C overnight. The next day, the coverslips were washed three times with DPBS and incubated for 60 minutes with a Alexa Fluor 488-conjugated anti-Rabbit IgG secondary antibody (**Table 2**). They were then washed three times with DPBS and once with distilled water. Finally, they were mounted onto glass slides using mounting solution containing the nuclear stain 4’,6-diamidino-2-phenylindole (Sigma, F6057). Samples were imaged on a Leica DMi8 microscope with a Leica DFC3000 G camera at 10× magnification.

**Table 2:** Antibodies used in this study.

Marker	Manufacturer	Reference Nr.	Dilution	Incubation	Host
Primary antibodies					
COL1	Abcam	ab138492	1:500	O/N 4 °C	Rabbit
SSEA4-AF488 conjugated	Invitrogen	53-8843-42	1:200	2 h, RT	Mouse
NANOG	Cell signalling	4903S	1:25	2 h, RT	Rabbit
SOX2	Milipore	AB5603	1:400	O/N, 4 °C	Rabbit
Secondary antibodies					
Anti-Rabbit-AF488	Invitrogen	A11008	1:500	1 h, RT	Goat
Anti-Rabbit-AF594	Invitrogen	A11072	1:500	1 h, RT	Goat

O/N, overnight; RT, room temperature; AF, Alexa Fluor™.

Image analysis was performed using the opensource software Fiji version 2.9.0 on Java 1.8.0 (46). A one-way nested ANOVA was performed to compare measurements obtained from healthy controls (C1-C5) against each patient group. For measuring collagen fibril coherency, the OrientationJ (47, 48) plugin was used. Coherency describes how consistently fibrils are aligned within a region of interest and is expressed as a fractional value. Low coherency values indicate that fibrils are randomly oriented in all directions and disorganised (isotropic), whereas high coherency values indicate that fibrils are consistently aligned in a common direction (anisotropic). For co-localization analysis of type I collagen with R-CHP, the Ridge Detection plug-in was used to generate binary masks for type I collagen and R-CHP images to exclude most non-fibrous structures resulting from the intracellular staining of unfolded collagen. Parameters used in the image analyses including saturation, coherency and empty grid thresholds, grid size for OrientationJ and minimum and maximum line width, low and high contrast values and minimum branch length for Ridge Detection were optimised by testing different values and selecting those that provided the best signal to noise ratio (refer to data repository Mask_Colocalization.ijm and parameters_collagen.txt for values used). This was followed by a pixel-by-pixel comparison of these two masks to locate overlapping pixels. A coefficient that represents the fraction of overlapping pixels compared to the total amount of positive pixels in the type I collagen mask is calculated and represented as percentage colocalisation.

### 2.3 Generation of fibroblast-derived induced pluripotent stem cells

Fibroblasts from a healthy control, a COL1A2-OI (p.Gly772Arg), a MBTPS2-OI (p.Asn459Ser), a MBTPS2-OI (p.Leu505Phe) and a MBTPS2-KFSD (p.Asn508Ser) (**Table 1**) between passage P4 and P6 were plated to achieve approximately 50% confluency for transduction with Sendai particles to deliver Yamanaka reprogramming factors (CytoTune iPSC2.0 kit, A16517, lot2190073). The following multiplicity of infection (MOI) were used: polycistronic Klf4–Oct3/4–Sox2 (MOI = 5), cMyc (MOI = 5), and Klf4 (MOI = 3). Cells were provided with fibroblast culture medium for the first 6 days. At 6 days post-transduction, cells were detached using trypsin and transferred onto 10-cm dishes (Sarstedt 83.3902) coated with Cultrex Reduced Growth Factor Basement Membrane Extract PathClear (BME, R&D Systems, 3433-010-01) at approximately 1mg/60cm^2^. The cells were then cultured in StemFlex medium (Gibco, A3349401). Between 19 and 29 days post-transduction, emerging colonies were transferred from the 10-cm onto individual wells of BME-coated 96-well plates. iPSC colonies were detached with Versene (Gibco, 15040066) and sequentially expanded onto BME-coated 24-well and 6-well plates. The StemFlex medium was supplemented with 1X RevitaCell™ Supplement (Gibco, A2644501) during passaging of the iPSCs. Four to six iPSC clones were subsequently used for further characterisation and differentiation experiments.

The rationale for selecting the COL1A2 p.Gly772Arg variant for further in-depth osteogenic characterisation and comparison with MBTPS2-associated OI was the well-defined clinical phenotype of the affected male patient (patient 7 in reference 5), which closely resembled that of the male patients with MBTPS2-OI. Shared clinical features included short stature, long bone deformities, pectus carinatum, and fractures with prenatal onset.

### 2.4 Characterization of iPSC

Fibroblasts and iPSC lines were analysed for chromosomal integrity by standard G-banding karyotyping to rule out aberrations including aneuploidy and translocations that could arise from cell reprogramming and long-term culture. Cells were cultured and harvested at ~70% confluency. Mitotic arrest was induced by treatment with 0.02 µg/mL colcemid (ThermoFisher, 15212012) for 45 minutes at 37 °C. Cells were subsequently dissociated using Accutase, subjected to hypotonic treatment with 0.075 M KCl (ThermoFisher, 10575090) for 10 minutes at 37 °C, and fixed in methanol:acetic acid (3:1, v/v). Fixed cell suspensions were dropped onto glass slides and air-dried. Chromosome spreads were stained using trypsin-Giemsa (G-banding). Metaphase spreads were analysed using light microscopy.

The expression of pluripotency markers was examined by immunocytochemistry (ICC) and quantitative reverse transcription polymerase chain reaction (qRT-PCR). ICC was performed by plating iPSCs on glass coverslips coated with poly-L-lysine and BME. The cells were fixed with 4% paraformaldehyde (as described above) at approximately 50% confluency. Specimens were permeabilised with 0.2% Triton X-100 (Sigma, X100) in DPBS for 15 minutes, incubated for 15 minutes with 100 mM Glycine (PanReac Applichem, A1067) and blocked with 1% BSA (Sigma, A3912) in DPBS at RT for 1 hour. The coverslips were then incubated overnight at 4 °C with primary antibodies against SOX2, NANOG and SSEA4 (**Table 2**). The next day, coverslips were washed three times with DPBS and incubated with Alexa Fluor 594-conjugated secondary antibodies (**Table 2**). The specimens were then washed three times with DPBS and once with distilled water, then mounted onto glass slides using Fluoroshield mounting medium containing 4’,6-Diamidin-2-phenylindol (Sigma, F6057). Fluorescence imaging was performed using a Leica DMi8 fluorescence microscope.

For qRT-PCR, RNA was harvested using RNeasy Mini Kit (QIAGEN, 74104) according to manufacturer’s instructions and reverse transcribed to cDNA using the High-Capacity RNA-to-cDNA Kit (Applied Biosystems, 4387406). qRT-PCR was performed using PowerTrackSYBR Green Master Mix (Applied Biosystems, A46012), the corresponding forward and reverse primers (**Table 3**, 400nM each), cDNA diluted to 5 ng/µl and nuclease-free water on a Quantstudio 7 Pro RealTime PCR System machine (Applied Biosystems). Gene expression levels were calculated relative to *GAPDH* using the ΔCt method with correction for primer efficiencies.

**Table 3:** Primers sequences used in qRT-PCR.

Target	Primer sequences (5’ – 3’)	Marker/function
*GAPDH*	Forward: GGCATCGACTGTGGTCATGAG Reverse: TGCACCACCAACTGCTTAGC	Endogenous control
*SOX2*	Forward: TCACATGTCCCAGCACTACC Reverse: CCCATTTCCCTCGTTTTTCT	iPSC
*POU5F1*	Forward: GAGGAGTCCCAGGACATCAA Reverse: CATCGGCCTGTGTATATCCC	iPSC
*NANOG*	Forward: CCAAATTCTCCTGCCAGTGA Reverse: CAGGTGGTTTCCAAACAAGAA	iPSC
*TBXT*	Forward: TGCTTCCCTGAGACCCAGTT Reverse: GATCACTTCTTTCCTTTGCATCAAG	Primitive streak
*TBX6*	Forward: AGCCTGTGTCTTTCCATCGT Reverse: GCTGCCCGAACTAGGTGTAT	Presomitic mesoderm
*MEOX1*	Forward: GGCAGCGTACCCTGACTTC Reverse: GGTCCCCATTTCCTTGGAACC	Somite
*PAX3*	Forward: AGCTCGGCGGTGTTTTTATCA Reverse: CTGCACAGGATCTTGGAGACG	Somite
*PAX9*	Forward: AGCAGGGTCATTACGACTCAT Reverse: CTGGGGTACGAGTAGATGTGG	Sclerotome
*RUNX2*	Forward: TTACTTACACCCCGCCAGTC Reverse: TATGGAGTGCTGCTGGTCTG	Osteoprogenitor
*ALPL*	Forward: GGAAGACACTCTGACCGTGG Reverse: GGGGGCCAGACCAAAGATAG	Pre-osteoblast
*COL1A1*	Forward: GGACACAGAGGTTTCAGTGGT Reverse: GCACCATCATTTCCACGAGC	Pre-osteoblast
*MSX2*	Forward: CGGAAAATTCAGAAGATGGAGCG Reverse: CGGCTTCCGATTGGTCTTGTGT	Osteoblast
*DLX5*	Forward: TACCCAGCCAAAGCTTATGCCG Reverse: GCCATTCACCATTCTCACCTCG	Osteoblast
*PHEX*	Forward: GAAGCCGACTACTTTGGCAACG Reverse: TGGTGGATGCACTGTAGAAGGC	Osteocyte
*SCD1*	Forward: CCTGGTTTCACTTGGAGCTGTG Reverse: TGTGGTGAAGTTGATGTGCCAGC	Fatty acid metabolism
*FADS1*	Forward: CTGTCGGTCTTCAGCACCTCAA Reverse: CTGGGTCTTTGCGGAAGCAGTT	Fatty acid metabolism
*FADS2*	Forward: TGCAACGTGGAGCAGTCCTTCT Reverse: GGCACATAGAGACTTCACCAGC	Fatty acid metabolism
*DHCR7*	Forward: TCCACAGCCATGSTGACCAATGC Reverse: CGAAGTGGTCATGGCAGATGTC	Sterol metabolism
*DHCR24*	Forward: CAGGAGAACCACTTCGTGGAAG Reverse: CCACATGCTTAAAGAACCACGGC	Sterol metabolism

### 2.5 Paraxial mesoderm and osteogenic differentiation

iPSCs were differentiated toward the osteoblast lineage using a stepwise differentiation protocol that was previously applied on human pluripotent stem cells (49). Five independent sets of differentiation experiments were performed, each using one iPSC clone per cell line. Overall, four clones from the control iPSC line and three clones from each patient-derived line were evaluated for their differentiation potential.

Sclerotome differentiation was initiated through replacement of the cell culture medium with basal differentiation medium (BDM) comprising DMEM/F-12 (Gibco, 11320074) supplemented with 1× Insulin-Transferrin-Selenium (Gibco, 41400045) and 0.5× penicillin–streptomycin (Gibco, 15140122). iPSCs were first directed toward a presomitic mesoderm (PSM) fate by supplementation of BDM with 3µM of Wnt agonist CHIR99021 (Sigma, SML1046) for two days. Subsequently, to direct the cells towards the nascent somite, the BDM was supplemented for another two days with 200nM LDN193189 (Stemcell Technologies, 72147) and 10µM SB431542 (Stemcell Technologies, 72234), which are antagonists of BMP and TBF-β pathways, respectively. On day 4 of the differentiation program, cells were dissociated and replated onto BME-coated 6-well plates at a density of 350,000 cells per well, followed by culture for an additional two days in BDM supplemented with 20ng/mL of Fibroblast Growth Factor 2 (FGF2, Stemcell Technologies, 78003) and 300nM of the Hedgehog signalling pathway activator Smoothened agonist (SAG, Tocris 5282) to generate sclerotome-like cells. During the differentiation process, cells were supplied daily with fresh medium containing the respective signalling pathway agonists or antagonists. Osteogenic differentiation of sclerotome cells was then performed over 4 weeks by replacing the medium with fresh StemPro Osteogenesis Differentiation Medium (Gibco, A1007201) every two to three days.

At defined time points (daily for iPSC to sclerotome differentiation, weekly for osteogenic differentiation), RNA was extracted from the differentiating cells for qRT-PCR using the method described above to follow the temporal expression of lineage specific markers (**Table 3**). Statistical analysis was performed using one-way ANOVA comparing the healthy control to each patient iPSC at each time point. During the osteogenic differentiation of sclerotome cells, cells were fixed weekly for assessment of extracellular matrix mineralisation by Alizarin Red staining (Sigma, 2003999). Stained wells were imaged using an Epson V39 II flatbed scanner. Images were analysed using Fiji (version 2.9.0 on Java 1.8.0). Briefly, images were converted to 8-bit grayscale, and the well area was defined as the region of interest (ROI). A fixed threshold was then applied to identify positively stained regions. Quantitative measurements included the percentage of positively stained area within the ROI and the sum of raw grey values (pixel intensities) within the thresholded regions.

## 3 Results

### 3.1 COL1 quality is affected in COL1-OI caused by glycine substitution but not COL1-haploinsufficiency OI, MBTPS2-OI and MBTPS2-KFSD

**Figure 1:**
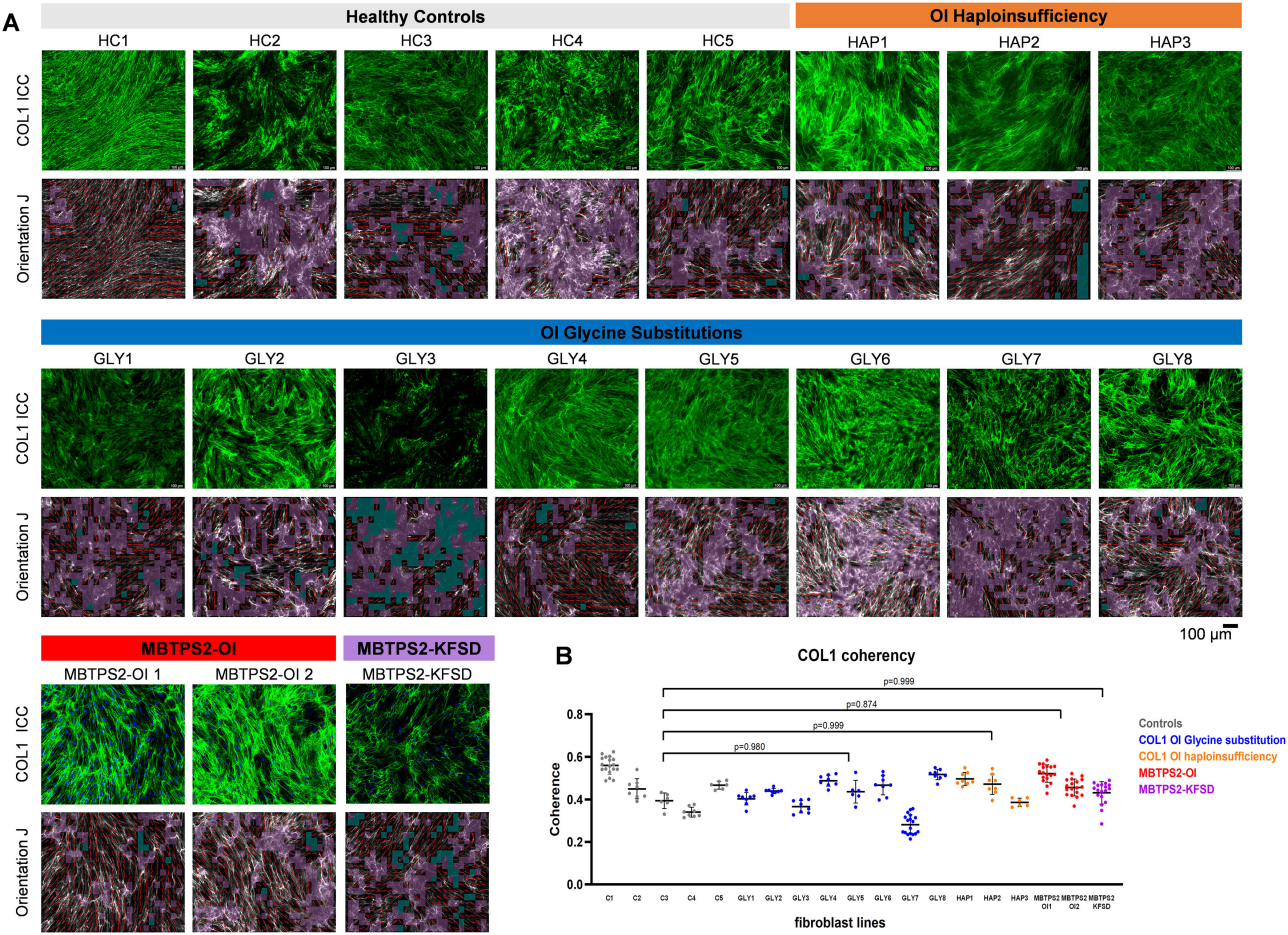
Extracellular type I collagen fibril synthesised by primary fibroblasts were analysed by immuno-staining and Fiji-based image analysis. **(A)** Representative images of type I collagen staining (COL1 ICC) and fibril orientation analysis (OrientationJ) are shown for each fibroblast line. For OrientationJ analysis, each image was divided into smaller grids. Grids with signal levels below threshold are represented in teal; purple-coloured grids indicate no dominant orientation and red vectors indicate the dominant fibril orientation within each grid. Scale bar 100 µm. **(B)** Each data point represents the average collagen I coherence value per image. A one-way nested ANOVA was performed to compare coherence of healthy controls (C1-C5) against each patient group.

The organisation of extracellular type I collagen fibrillar network produced by fibroblasts was assessed by ICC staining of collagen type I. Representative images for each fibroblast line are shown in the top panels of **Figure 1A**. To assess local fibril orientation using OrientationJ, each image was divided into smaller grids. Grids with signal levels below a pre-defined threshold are represented in teal; purple-coloured grids indicate that no dominant orientation could be determined. The red vectors indicate the dominant fibril orientation within each grid (**Figure 1A**, bottom panels). The average coherence value per image was calculated and plotted as a single data point; intragroup variability was observed for the coherency of extracellular type I collagen, with no significant differences between the control group and individual patient groups or single patient sample for the single MBTPS2-KFSD case (**Figure 1B**).

**Figure 2:**
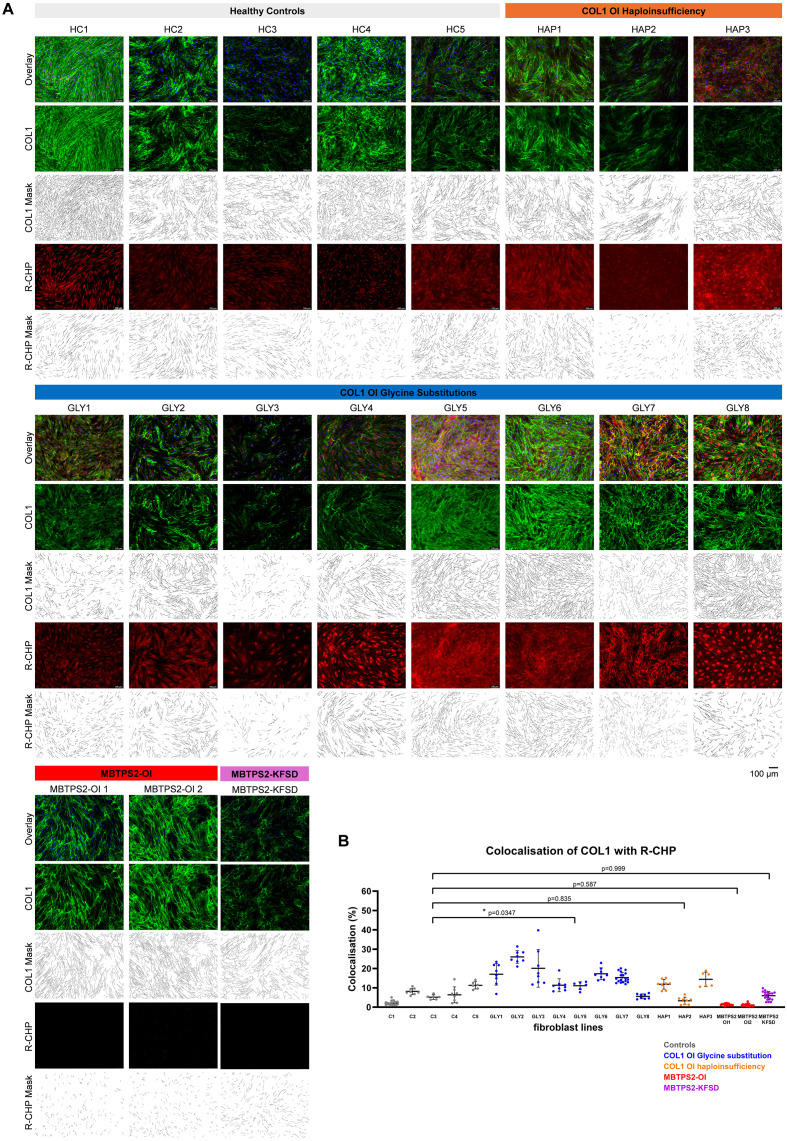
The quality of extracellular collagen synthesised by primary fibroblasts were analysed by co-staining with R-CHP and antibodies against type I collagen, followed by Fiji-based image analysis. **(A)** Representative images of type I collagen staining (COL1, green) and R-CHP staining (red) and their overlay images are shown for each fibroblast cell line. Binary masks for COL1 and R-CHP were generated by Ridge Detection and compared to quantify the proportion of overlapping pixels. Scale bar 100 µm. **(B)** The proportion of positive pixels in COL1 masks that overlaps with positive pixels in the corresponding R-CHP masks were calculated for each image. Each data point represents one image. A one-way nested ANOVA was performed to compare the amount of COL1 colocalising with R-CHP between healthy controls (C1-C5) and each patient group.

Misfolded type I collagen was assessed by co-staining for type I collagen with a specific antibody (**Figures 2A**, green) and R-CHP, which binds unfolded and misfolded triple helical regions in all collagen types (**Figures 2A**, red). Because collagen molecules are not fully folded during intracellular biosynthesis, membrane-permeable R-CHP binds intracellular unfolded collagens in addition to extracellular misfolded collagen fibrils. Therefore, Ridge Detection was applied using Fiji to generate binary masks that preferentially selected extracellular fibrillar collagen for subsequent colocalisation analyses. The proportion of type I collagen that colocalised with R-CHP staining was significantly elevated in COL1-OI fibroblasts with glycine substitutions in the triple helical region (GLY) compared to healthy controls (**Figure 2B**). In contrary, there were no significant differences in the colocalisation of type I collagen with R-CHP in the COL1-OI haploinsufficiency and MBTPS2-OI groups, nor in the MBTPS2-KFSD fibroblasts, compared to controls (**Figure 2B**). The image analyses were performed in two independent experiments for C1, GLY7, MBTPS2-OI1, MBTPS2-OI2 and MBTPS2-KFSD to assess the technical reproducibility of the analysis pipeline. The overall trend and biological effect of the genetic variants are maintained, despite minor inter-experiment variations (**Supplementary Figure 1**).

Overall, these data indicate that collagen triple-helix folding was severely disrupted specifically in OI caused by glycine substitutions in COL1A1 or COL1A2, whereas fibril assembly appeared unaffected across all patient groups under our experimental conditions.

### 3.2 Differential mineralisation patterns among genetic forms of OI despite preserved osteogenic potential

**Figure 3:**
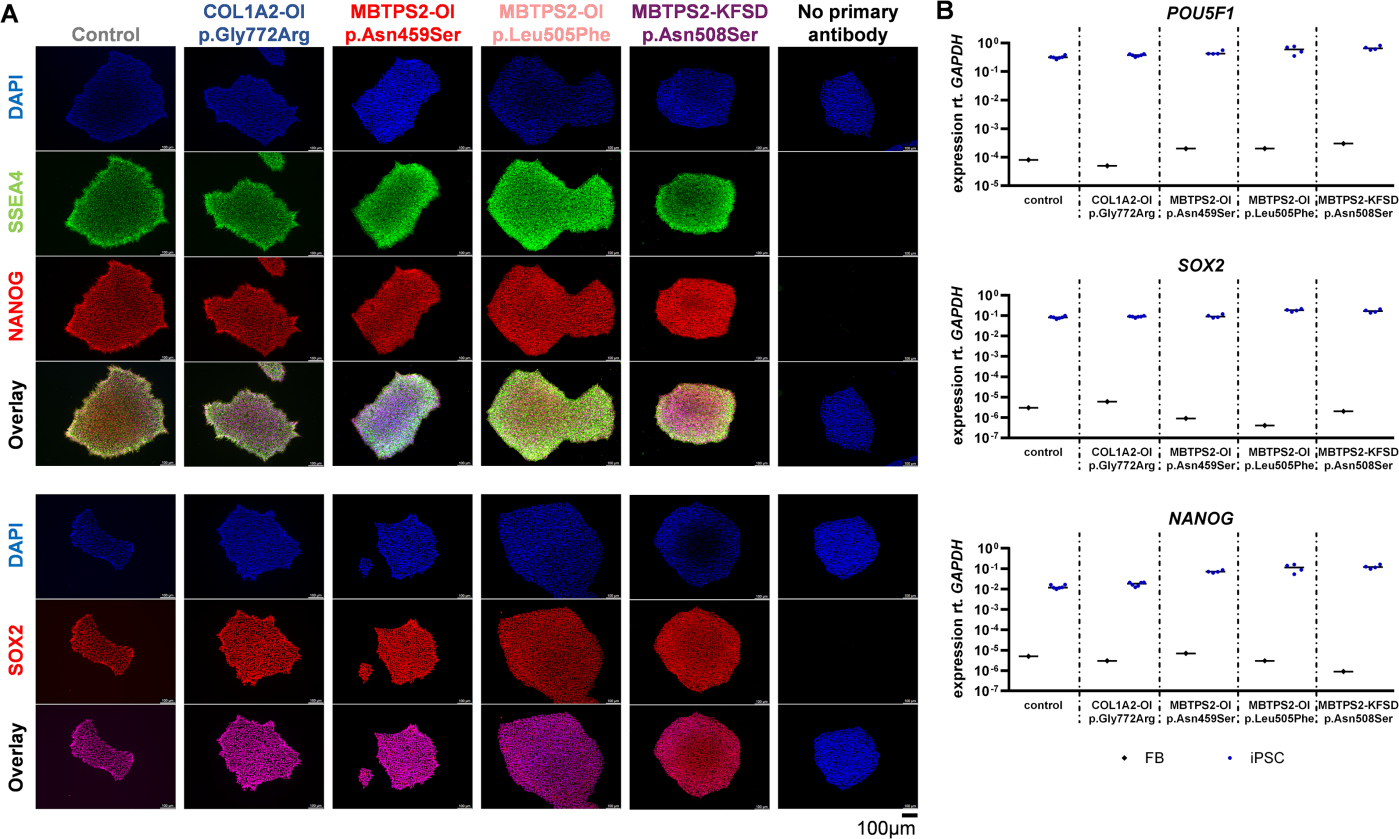
The protein and gene expression of pluripotency markers were characterised in iPSCs. **(A)** Protein expression of pluripotency markers SSEA4 (green), NANOG (red, top panel) and SOX2 (red, bottom panel) was analysed by ICC. Representative images are shown for each iPSC line. Scale bar 100 µm. **(B)** Gene expression of pluripotency markers *POU5F1*, *SOX2*, and *NANOG* relative to endogenous control *GAPDH* was quantified in patient-derived fibroblasts (black) and the reprogrammed iPSCs (blue, each data point represents one iPSC clone) by qRT-PCR.

All five control- and patient-derived fibroblasts were successfully reprogrammed into iPSCs, as demonstrated by the expression of the pluripotency markers SSEA4, NANOG, and SOX2 in iPSC colonies (**Figure 3A**), as well as by the elevated gene expression of *POU5F1*, *SOX2* and *NANOG* in iPSCs compared to their parental fibroblasts (**Figure 3B**). Karyotype analysis confirmed the absence of chromosomal abnormalities in the donor fibroblasts, with all five samples exhibiting a normal male karyotype (46,XY). Chromosomal integrity was preserved in iPSCs at passages P10-15 (**Supplementary Figure 2**).

**Figure 4:**
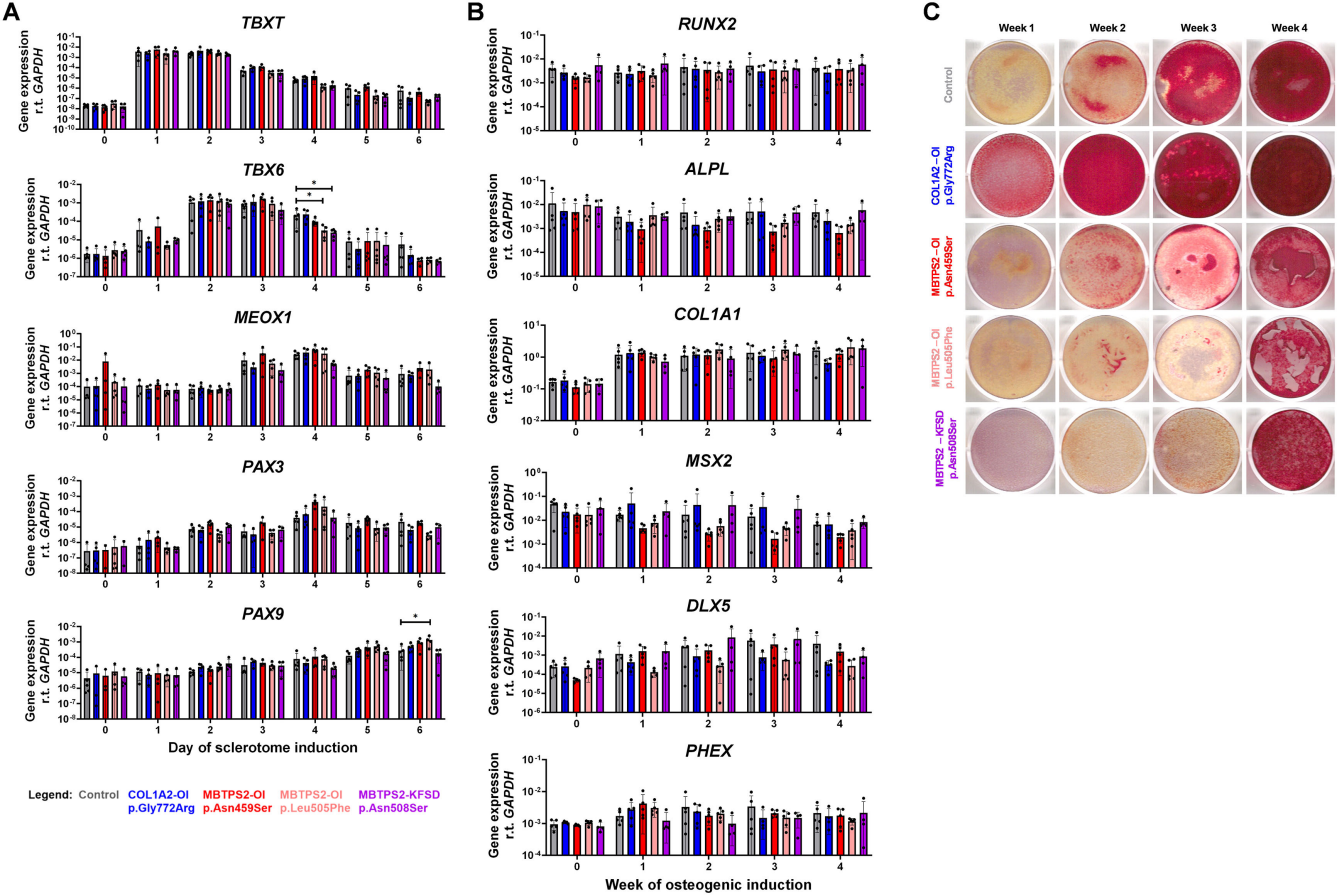
Stepwise differentiation of iPSCs into sclerotome, followed by 4 weeks of osteogenic differentiation, was performed and characterised by quantifying lineage-specific gene expression and extracellular matrix mineralisation. **(A)** Gene expression analysis of markers of the primitive streak (*TBXT*), presomitic mesoderm (*TBX6*), somite (*MEOX1* and *PAX3*) and sclerotome (*PAX9*) was performed daily during stepwise differentiation of iPSCs to sclerotome. **(B)** Gene expression analysis of markers of osteoprogenitor (*RUNX2*), pre- and early osteoblast (*ALPL* and *COL1A1*), osteoblast (*MSX2* and *DLX5*) and osteocyte (*PHEX*) was performed weekly during osteogenic differentiation. Statistical analysis was conducted using one-way ANOVA comparing the healthy control to each patient iPSC at each time point (* p<0.05). **(C)** Alizarin red staining was performed weekly to monitor the mineralisation pattern during osteogenic differentiation.

Temporal expression of key developmental markers during iPSC-to-sclerotome differentiation was monitored daily by qRT-PCR. Expression of the primitive streak marker *TBXT* increased rapidly upon initiation of sclerotome induction, peaking at days 1 and 2. This was followed by increased expression of the posterior PSM marker *TBX6*, which peaked on days 2 and 3. Subsequently, the expression of somite-associated markers *MEOX1* and *PAX3* peaked on day 4, while expression of the sclerotome marker *PAX9* increased steadily until day 6 of induction (**Figure 4A**). At the time points (days) of peak gene expression, no differences in expression levels were observed among the cell lines. The stage-specific changes in gene expression were consistently observed across all iPSC lines, illustrating the sequential progression of differentiation and confirming successful induction of sclerotome-like cells in both control and patient-derived cell lines. Only minor differences in gene expression relative to control were observed for *TBX6* in MBTPS2-OI p.Leu505Phe and MBTPS2-KFSD p.Asn508Ser on day 4, and for PAX9 in MBTPS2-OI p.Leu505Phe cells on day 6 of sclerotome induction. As these differences were transient and not consistently observed, they likely suggest that the overall differentiation potential toward the sclerotome lineage is not compromised in patient-derived iPSC lines.

Osteogenic differentiation of sclerotome-derived cells was further monitored over four weeks by weekly quantification of expression levels of key osteogenic marker genes. Upon osteogenic induction, *COL1A1* expression increased approximately 10-fold in all iPSC lines and remained relatively stable throughout the four-week period, indicating a transition from osteoprogenitors to mature osteoblasts. Expression of osteoblast marker *DLX5* and osteocyte marker *PHEX* gradually increased over time, whereas osteoprogenitor marker *RUNX2*, and pre- and early osteoblast markers *ALPL* and *MSX2* expression levels remained largely constant during osteogenic induction (**Figure 4B**). No significant differences in gene expression level were observed among the cell lines at any time point for any of the analysed genes. Compared to previous studies using the same differentiation protocol with human embryonic stem cells as the starting material (49), the transcriptional response to osteogenic induction appeared dampened in fibroblast-derived iPSCs used in the present study. Nonetheless, Alizarin Red staining demonstrated progressively increased mineralisation over the 4-week osteogenic induction period, indicating successful osteogenic differentiation (**Figure 4C**).

Interestingly, distinct mineralisation patterns were observed depending on the genetic cause of disease. Compared to control cells, ECM hypermineralisation was evident in COL1A2-OI cells, with positive Alizarin Red staining detectable after only one week of osteogenic induction and more intense staining than controls at week 4. In contrast, ECM hypomineralisation was observed in cells carrying MBTPS2 variants associated with either OI or KFSD (**Figure 4C**). Partial detachment of MBTPS2-OI p.Leu505Phe cells was observed at week 4; however, Alizarin Red staining remained less intense than in controls in regions where cells remained attached. As no significant differences in the expression of pre-osteoblast or osteoblast marker genes were observed between the cell lines at any assessed time point, we infer that osteogenic differentiation was largely preserved. Instead, the data suggest that the primary defect likely lies in the mineralisation process rather than in the differentiation of osteogenic cells. A summary of the experimental workflow and diverging pathological mechanisms underlying MBTPS2-OI and classical OI caused by glycine substitutions in type I collagen is depicted in **Supplementary Figure 4**.

### 3.3 Mild effects on fatty acid metabolism

**Figure 5:**
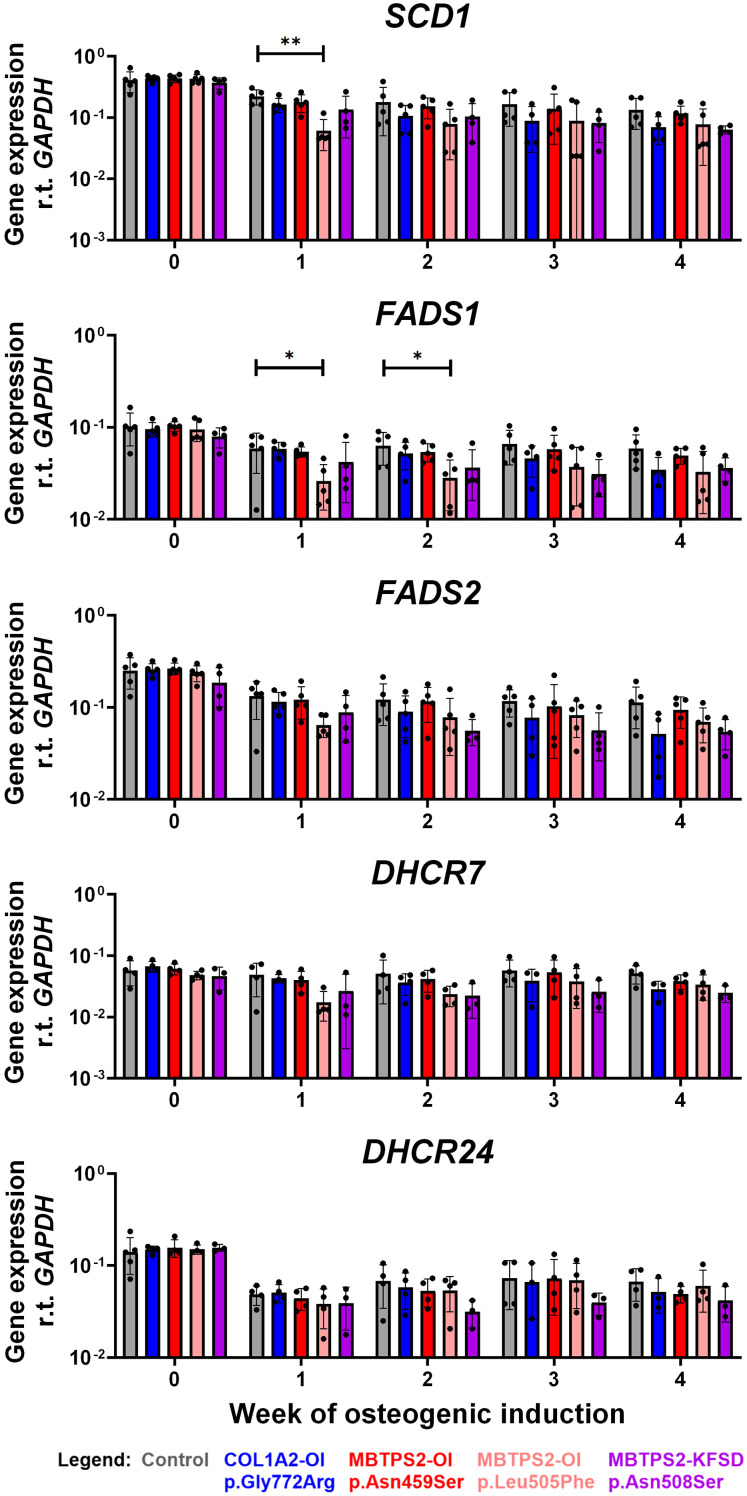
qRT-PCR analysis of genes involved in fatty acid (*SCD1*, *FADS1* and *FADS2*) and sterol (*DHCR7* and *DHCR24*) metabolism was performed weekly during osteogenic differentiation. Statistical analysis was conducted using one-way ANOVA comparing the healthy control to each patient iPSC at each time point (*p<0.05, **p<0.01).

We previously reported reduced expression of SREBP-regulated genes involved in lipid metabolism, as well as altered fatty acids abundance ratios in fibroblasts derived from *MBTPS2*-OI patients (21). Here, the expression of these SREBP-regulated genes was investigated following osteogenenic induction of iPSC-derived sclerotome cells. The trends previously observed in fibroblasts could only be partially recapitulated in the cells undergoing osteogenesis, with subtle repression of *SCD1* and *FADS1* expression observed in *MBTPS2*-OI p.Leu505Phe cells (**Figure 5**).

## 4 Discussion

In the last two decades, a large body of knowledge on OI pathophysiology has been generated using patient-derived primary fibroblasts and animal models, including mice, zebrafish and dogs. Together, these models capture many of the molecular signatures, phenotypic features, and severity levels of both dominant and recessive forms of OI (50). Animal models provide the opportunity to examine OI in both control and affected animals matched for age, sex, and genetic background, which is not possible with patient-derived material alone. Murine models remain the most extensively used and include the type I collagen-related *oim* mouse, Brtl and Amish/G610C knock-in lines, as well as models of rarer recessive forms of OI targeting genes such as Crtap, P3h1/Lepre1 and Ifitm5 (50, 51). Zebrafish models, including the Chihuahua mutant (52), more recently engineered lines with collagen defects (53), and crispant fish (26), are valuable because they display key skeletal hallmarks of OI and enable rapid phenotypic assessment and scalable drug screening. Furthermore, zebrafish bone mutants tend to survive into adulthood more readily than corresponding mouse models and have the unique ability to regenerate various tissues, including the caudal fins, thereby allowing investigation of tissue patterning and signalling pathways involved in bone regeneration (53, 54).

However, available animal models also have important limitations for modelling human bone disease. No single model can capture the full genetic and phenotypic heterogeneity of OI, and rarer genetic forms remain under-represented. In addition, the phenotype of animal models do not always recapitulate those observed in patients. Examples include *CRTAP* and *LEPRE1* variants, which cause severe to lethal OI in humans (55, 56) whereas the corresponding mouse models present milder phenotypes (57, 58). Similarly, the *IFITM5* c.-14C>T variant is associated with moderate to severe OI in humans and increased mineralisation *in vitro* (59, 60), but results in perinatal lethality and decreased mineralisation in mice (61). In addition, teleost-specific whole-genome duplication complicates the use of zebrafish for modelling human genetic diseases, including collagen-related disorders with duplicated orthologues, and may obscure genotype–phenotype interpretation (54, 62). Furthermore, interspecies differences in bone tissue architecture exist (63, 64), contributing to substantial variability in therapeutic responses across models. Collectively, these limitations support the need for complementary disease models using human patient-derived systems to dissect disease mechanisms in genetically distinct forms of OI.

In this study, we adapted a previously published differentiation protocol by Xi et al. (49) to induce osteogenic differentiation from fibroblast-derived iPSCs. While the original protocol demonstrated robust upregulation of osteogenic markers and efficient osteoblast generation from pluripotent human embryonic stem cells (ESCs), our results showed comparatively limited induction of osteogenic gene expression in our iPSCs. A key distinction between the two systems is the starting cell type, which likely underlies the observed discrepancies. One plausible explanation for the reduced osteogenic differentiation efficiency is the persistence of epigenetic memory in iPSCs derived from somatic cells. Residual DNA methylation patterns and chromatin states from the donor fibroblasts can bias differentiation potential and restrict lineage transitions (65), in contrast to ESCs which lack epigenetic priming. Consistent with this, differences in transcriptomic profiles have been reported between ESCs and iPSCs-derived cells. For example, mouse iPSC-derived osteoblasts exhibit weaker induction of *Col1a1* and *Col1a2* than ESC-derived cells (66).

In OI associated with glycine substitution in type I collagen, the initiating defect lies within the matrix protein itself. As expected, a larger proportion of misfolded type I collagen was observed in COL1-OI patient samples with glycine-substitution variants, indicating a qualitative defect in the ECM deposited by the fibroblasts. In contrast, *COL1A1* variants causing frameshifts or premature stop codons are predicted to result in haploinsufficiency, which is associated with a quantitative rather than a qualitative defect in type I collagen.

Using our iPSC-derived osteoblast model, we observed hypermineralisation *in vitro* in COL1A2 p.Gly772Arg OI, consistent with earlier studies demonstrating increased bone mineralisation density distribution in bone explants from COL1-OI patients (67, 68). A key limitation in our ECM image analysis pipeline lies in image resolution and the difficulty of detecting thin fibrils using Ridge Detection, leading to potential under-detection of stained structures in the segmentation masks. The collagen image analysis workflow may be further optimised by higher resolution imaging of the specimens with confocal microscopy and by adapting various parameters defined in the Fiji macro for specific user requirements, such as for saturation, contrast, line width and minimum branch length. Nevertheless, our image analysis pipeline indicated that the quality of type I collagen appears preserved in fibroblasts from OI patients with COL1 haploinsufficiency compared with healthy controls, supporting the ability of the method to distinguish between COL1 structural defects (glycine substitutions) and quantitative defects caused by haploinsufficiency.

The quality of type I collagen appears unaffected in MBTPS2-OI, as demonstrated by a lack of significant changes in R-CHP signal and COL1 colocalisation. Furthermore, hypomineralisation was observed in osteoblasts derived from iPSCs of both genetic variants of MBTPS2-OI. Although this study is limited by the absence of isogenic control cell lines and the small number of biological replicates, which restrict the generalisability of the findings, the results nevertheless indicate that the molecular signatures and pathogenic mechanisms underlying MBTPS2-OI appears fundamentally different from the primary structural collagen defects that underlie classical OI caused by COL1A1 and COL1A2 glycine substitutions. This distinction highlights that OI is ultimately a genetically heterogenous clinical disease with multiple underlying causes and a spectrum of distinct pathomechanisms that converge on a shared clinical phenotype of bone fragility. Accordingly, therapeutic development and clinical management of patients should take the specific genetic form of the disease in order into account to achieve optimal outcomes.

Despite demonstrating distinct pathologies underlying MBTPS2-OI compared with classical COL1-OI, the mechanisms underlying MBTPS2-OI and its distinction from MBTPS2-IFAP/KFAD remain unclear. MBTPS2-KFSD iPSC-derived osteoblasts displayed hypomineralisation, a pattern similar to that of MBTPS2-OI, despite the absence of overt bone fragility in IFAP/KFSD patients. A plausible explanation for this apparent discrepancy is that the *in vitro* mineralisation assay is performed under simplified, cell-autonomous culture conditions, whereas bone mineral density *in vivo* reflects the integrated effects of bone formation and resorption involving multiple cell types, endocrine regulation by circulating hormones, mechanical loading and systemic physiological regulation of calcium and phosphate levels.

We previously described increased misfolded collagens deposited in the ECM by MBTPS2-OI patient fibroblasts, detected by R-CHP hybridisation (20); however, these R-CHP signals do not colocalise with type I collagen antibody-specific staining, suggesting that additional, as yet unknown, mechanisms contribute to impaired synthesis of collagenous proteins and dysregulation of ECM production in MBTPS2-OI. A limitation of this study is the restricted range of osteogenic readouts. Additional analyses, such as comprehensive proteome profiling of the ECM and assessment of the biomechanical properties of the *in vitro* cultures, could provide further insights into the distinct pathogenic mechanisms underlying MBTPS2-OI compared with classical type I collagen forms of the disease. We further propose that future mechanistic studies should model chondrogenesis using patient-derived iPSCs to more faithfully recapitulate the process of endochondral ossification during bone development.

## Data Availability

The data and image analysis pipeline method presented in the study are deposited in the Zenodo repository, accession number 10.5281/zenodo.19884590.

## Supplementary material

Supplementary Figure 1 Image analyses of type I collagen fibril orientation and colocalisation with R-CHP were performed in two independent technical replicates for control, COL1A2 p.Gly772Arg, MBTPS2-OI p.Asn459Ser, MBTPS2-OI p.Leu505Phe and MBTPS2-KFSD p.Asn508Ser (corresponding to C1, GLY7, MBTPS2-OI1, MBTPS2-OI2 and MBTPS2-KFSD, respectively, in **Figures 1** and **2**). Each group of data points represents an independent technical replicate, and each data point represents the measurement for a single image.

Supplementary Figure 2 Karyotyping analysis of the patient fibroblasts and three iPSC clones per line was performed to confirm maintenance of chromosomal integrity. A representative karyotype is shown for each fibroblast and one iPSC clone per line.

Supplementary Figure 3 Images of Alizarin red-stained wells were analysed using Fiji. **(A)** A fixed threshold was applied to identify positively stained regions, marked in white. **(B)** The percentage of positively stained area and Raw Integrated Density (RawIntDen) representing the sum of all raw grey values in arbitrary units (a.u.) were measured.
